# Prognostic significance of pathological complete response following neoadjuvant chemotherapy for operable breast cancer

**DOI:** 10.3892/ol.2014.1792

**Published:** 2014-01-14

**Authors:** HIDEMI KAWAJIRI, TSUTOMU TAKASHIMA, NAOKI AOMATSU, SHINICHIRO KASHIWAGI, SATORU NODA, NAOYOSHI ONODA, TETSUROU ISHIKAWA, KOSEI HIRAKAWA

**Affiliations:** Department of Surgical Oncology, Osaka City University Graduate School of Medicine, Osaka 545-8585, Japan

**Keywords:** breast cancer, pathological complete response, neoadjuvant chemotherapy, triple-negative subtype

## Abstract

The aim of the present retrospective study was to ascertain the significance of pathological complete response (pCR) on overall survival (OS) and disease-free survival (DFS) in each disease subtype of operable breast cancer. Using a single-institution database, 90 patients were identified, who received neoadjuvant chemotherapy (NAC) for operable breast cancer and were eligible for the analysis. In total, 10 patients (11.1%) had succumbed to their diseases and 20 (22.2%) had succumbed to their diseases or exhibited recurrences. The OS of patients with triple-negative (TN) tumors was significantly lower than that of patients with other disease subtypes (P=0.016). The DFS of patients with luminal tumors was higher than that of patients with other subtypes. Survival was improved with pCR following NAC (P=0.044). Across all subtypes, patients who achieved pCR exhibited a higher DFS than patients who did not, but not significantly. pCR only improved OS and DFS in the TN disease subtype (P=0.022 and P=0.048, respectively). pCR following NAC may have prognostic value in TN breast cancer.

## Introduction

Pathological complete response (pCR) to neoadjuvant chemotherapy (NAC) for breast cancer is a significant predictor of overall survival (OS) and disease-free survival (DFS) (1–3). However, Ring *et al* previously reported that pCR improves prognosis in patients with estrogen receptor (ER)-negative tumors, but not in patients with ER-positive tumors (4). In addition, Fasching *et al* reported that patients with human epidermal growth factor receptor 2 (HER2)-negative disease have a more favorable prognosis when pCR is achieved, despite the marked proliferation potential of these tumors based on Ki-67 staining (5). von Minckwitz *et al* reported that pCR is a suitable surrogate endpoint in the luminal B/HER2-negative, HER2-positive (non-luminal) and triple-negative (TN) subtypes, but not in the luminal B/HER2-positive or luminal A subtypes. No correlation was observed between pCR and prognosis in ER-positive tumors, including luminal A tumors (6). Although the authors reported that pCR has prognostic value in intrinsic subtypes, the study combined patients who had received various chemotherapeutic regimens.

We previously reported the effect of NAC with FEC100 (fluorouracil/doxorubicin/cyclophosphamide) followed by weekly paclitaxel treatment, which is the standard regimen at Osaka City University Graduate School of Medicine (Osaka, Japan) (7). This regimen reaches a pCR rate of 38.9%. The addition of trastuzumab increases the pCR rate when HER2-positive patients are treated with sequential neoadjuvant paclitaxel and FEC chemotherapy (66.7%) (8). The objective of the present retrospective study was to assess the impact of the pCR achieved by this effective regimen in each subtype of breast cancer.

## Materials and methods

### Patient selection

In total, 90 females with histologically confirmed invasive ductal carcinoma of the breast and stage IIA to IIIA disease were included. All patients received NAC as initial treatment at our institution between November 2005 and February 2010. For each patient, the histological diagnosis was determined by core needle biopsy of the tumor. ER, progesterone receptor (PgR) and HER2 overexpression were evaluated in biopsy specimens prior to treatment.

Intrinsic subtypes were determined according to ER, PgR and HER2 status and not according to the pre-chemotherapy Ki-67 index and histological grade, since it was not possible to refer to these statuses from the disease profile. The luminal subtype was defined as ER-positive and/or PgR-positive and HER2-negative. The luminal HER2 subtype was defined as ER-positive and/or PgR- and HER2-positive. The HER2 subtype was defined as ER- and PgR-negative and HER2-positive, and the TN subtype was defined as ER-, PgR- and HER2-negative. As the luminal HER2 subtype included only six cases, ER- and/or PgR-positive tumors were included in the luminal subtype regardless of HER2 status in specific analyses.

### Treatment

Chemotherapy was administered in the ambulatory treatment center prior to locoregional therapy. The FEC100 regimen consisted of intravenous administration of fluorouracil (500 mg/m^2^; Kyowa Hakko Bio Co., Ltd., Tokyo, Japan), cyclophosphamide (500 mg/m^2^; Shionogi Co., Ltd., Osaka, Japan) and epirubicin (100 mg/m^2^; Pfizer, New York, NY, USA) every 21 days for four cycles, followed by weekly intravenous infusion of paclitaxel (80 mg/m^2^; Bristol-Myers, New York, NY, USA) for 12 weeks. HER2-positive patients received 2 mg/kg trastuzumab (4 mg/kg initially; Chugai Pharmaceutical Co., Ltd., Tokyo, Japan) concurrently with paclitaxel after February 2008.

Surgery (total or partial mastectomy with axillary lymph node dissection or sentinel node biopsy) was scheduled 3–6 weeks following the completion of NAC. All patients treated with partial mastectomy received postoperative whole breast irradiation; medial and lateral tangent fields with a total dose of 50 Gy divided into 25 fractions. In cases of close margins, the tumor bed was treated with an additional 10 Gy in five fractions with an electron beam. Regional nodal irradiation to the supraclavicular and chest wall was used in patients who received mastectomy with four or more positive lymph nodes.

Following the completion of systemic and local therapy, patients with ER- or PgR-positive tumors received tamoxifen (20 mg) or anastrozole (1 mg) daily according to menstrual status for 5 years. Patients with HER2-positive tumors received trastuzumab weekly (2 mg/kg) or tri-weekly (6 mg/kg) for 1 year.

### Pathological evaluation

Surgical specimens were fixed in buffered formalin and sectioned into 5-mm-thick slices to prepare paraffin-embedded blocks. The specimens were examined by pathologists following conventional hematoxylin/eosin staining. Pathological observations were evaluated in accordance with the 16th edition of the General Rules for Clinical and Pathological Recording of Breast Cancer from the Japan Breast Cancer Society (9). pCR was defined as no evidence of residual invasive cancer in the breasts and lymph nodes, including in patients with non-invasive or *in situ* cancer and in patients whom no residual cancer cells were identified.

### Immunohistochemistry (IHC) and fluorescence in situ hybridization (FISH)

All tumors were assessed for ER and PgR expression by IHC. Patients were classified as having ER- and/or PgR-positive tumors when >10% of cancer cells showed positive staining by IHC. HER2/neu expression was also assessed by IHC according to the 2007 ASCO/CAP Guidelines (10); if the IHC score was 3+, the specimen was considered to exhibit HER2 overexpression and if the score was 2+, FISH was performed to confirm gene amplification (>2.2-fold increase in fluorescence compared with that in the centromere).

### Follow-up

Patients were examined following each cycle of chemotherapy to evaluate clinical responses and adverse events. Following surgery or radiotherapy, patients visited every 3 months for 2 years, then every 3–6 months for the next 3 years. Beyond 5 years, patients were assessed annually by physical examination, mammography, ultrasonography, contrast-enhanced computed tomography and bone scan.

### Statistical methods

The correlation between each baseline clinical variable and the probability of achieving pCR was tested by univariate analysis using the χ^2^ or Fisher’s exact tests. The independent significance of each variable was assessed by multivariate logistic regression analysis using a step-up procedure. The odds ratios of pCR were calculated from the final model. OS and DFS were measured between the date of the surgery and mortality or recurrence, respectively; mortalities without recurrence were censored in the DFS analysis. Survival curves were calculated by the Kaplan-Meier method and differences were assessed by the log-rank test. SPSS 19.0 (SPSS, Inc., Chicago, IL, USA) was used for all statistical analyses. P<0.05 was considered to indicate a statistically significant difference.

## Results

### Clinical variables

Individual clinical variables are shown in Table I. Between November 2005 and February 2010, 90 patients at our institute received surgery following NAC. The age of the patients ranged between 26 and 71 years (median, 53 years) and the tumor sizes ranged between 15 and 60 mm (median, 30 mm). Follow-up time ranged from 8 to 83 months (median, 53 months) and clinical stages prior to NAC ranged from 2A to 3A.

pCR was observed in 38 of the 90 (42%) patients. By univariate analysis, T1 (P=0.013), ER-negative status (P=0.028) and PgR-negative (P=0.029) status were found to significantly correlate with pCR. In addition, tumor subtype was found to significantly correlate with pCR (P=0.049). Axillary lymph node involvement was associated with pCR, but the association did not reach statistical significance (P=0.058). Differences in pCR according to HER2 status and age were not statistically significant.

### OS and DFS

**Figure 1:**
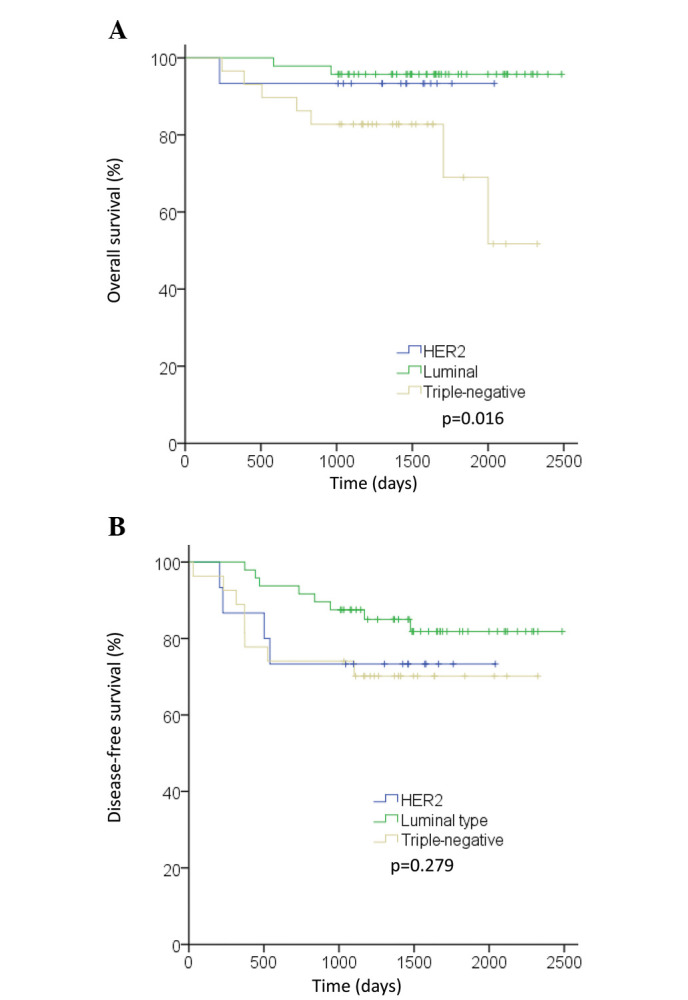
Kaplan-Meier (A) overall survival and (B) disease-free survival according to breast cancer subtype. HER2, human epidermal growth factor receptor 2.

At the median follow-up time of 53 months, 10 of the 90 (11.1%) patients had succumbed to their diseases and 20 of the 90 (22.2%) patients exhibited recurrences or had succumbed to their diseases without recurrences. In total, three of the 48 (6.3%) patients with the luminal subtype, six of the 27 (22.2%) patients with the TN subtype and one of the 15 (6.7%) patients with the HER2 subtype succumbed to their diseases. OS differed between subtypes (P=0.016). The prognosis of patients with TN tumors was significantly poorer than that of patients with other tumor subtypes (Fig. 1A). In total, eight of the 48 (16.7%) patients with the luminal subtype, eight of the 27 (29.6%) patients with the TN subtype and four of the 15 (26.7%) patients with the HER2 subtype exhibited recurrences or succumbed to their diseases without recurrences. Patients with the luminal subtype exhibited fewer DFS events than patients with other subtypes, but the difference was not statistically significant (Fig. 1B).

### Prognostic impact of pCR

**Figure 2:**
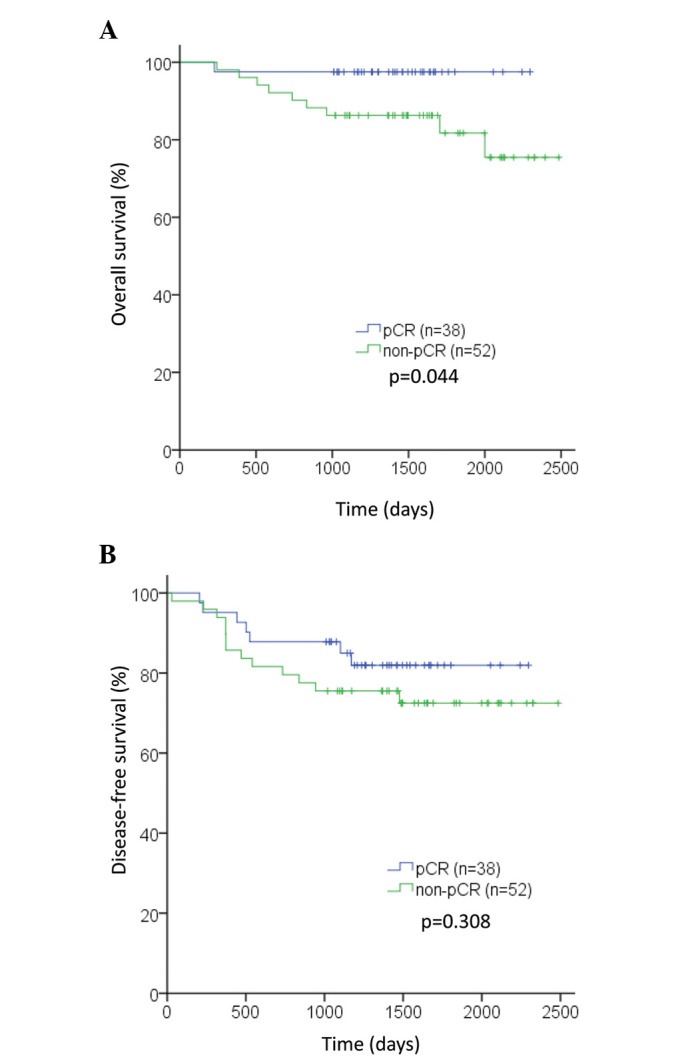
Kaplan-Meier prognostic impact of pCR on (A) overall survival and (B) disease-free survival. pCR, pathological complete response.

**Figure 3:**
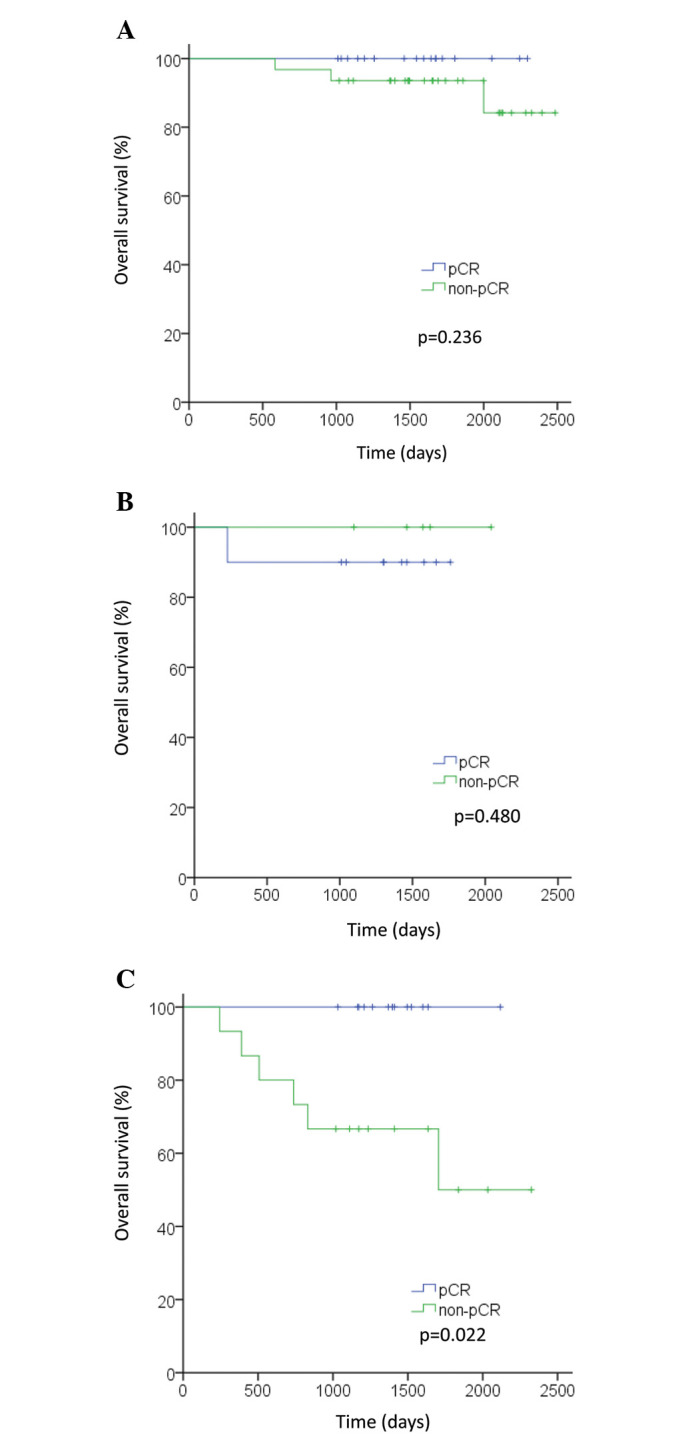
(A) Kaplan-Meier prognostic impact of pCR on overall survival in the (A) luminal, (B) human epidermal growth factor receptor 2 and (C) triple-negative disease subtypes. pCR, pathological complete response.

**Figure 4:**
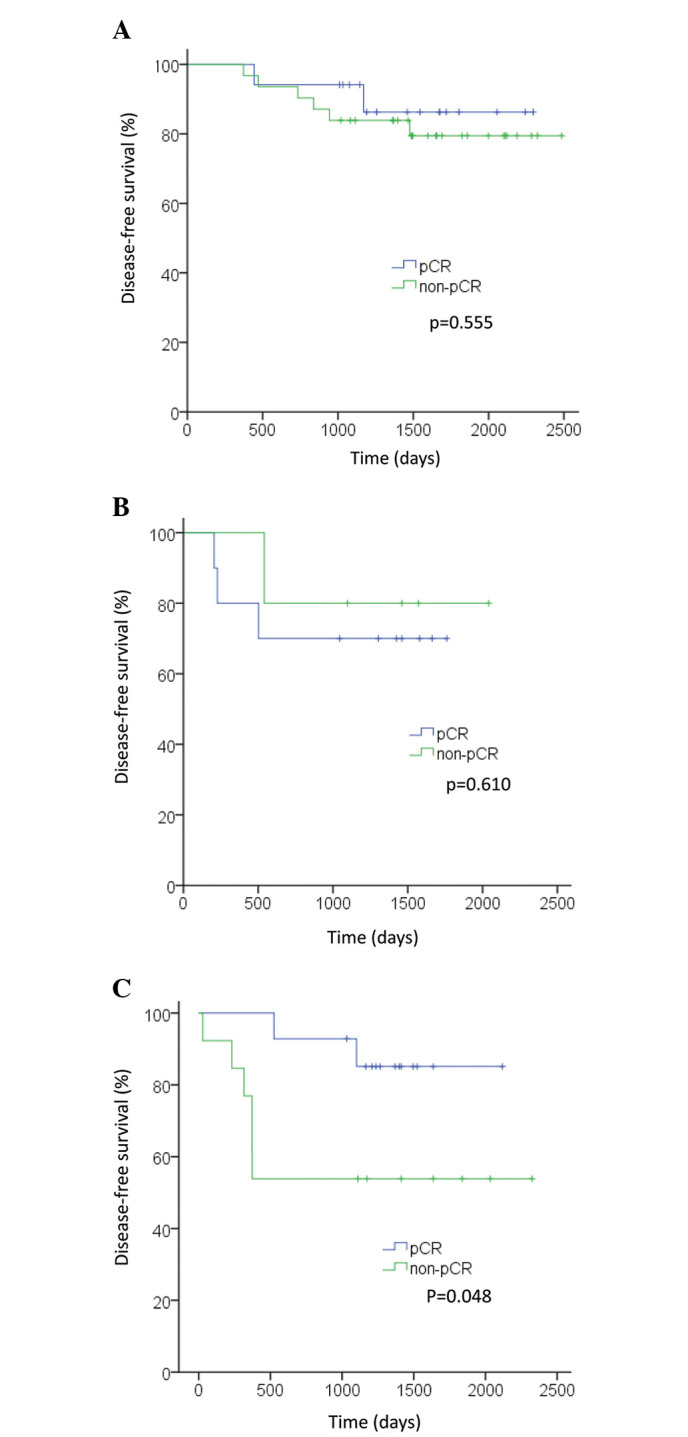
Kaplan-Meier prognostic impact of pCR on disease-free survival in the (A) luminal, (B) human epidermal growth factor receptor 2 and (C) triple-negative disease subtypes. pCR, pathological complete response.

pCR following NAC improved survival (P=0.044; Fig. 2A), but not DFS (P=0.31; Fig. 2B). In the TN subtype, patients who achieved pCR exhibited improved OS and DFS compared with patients who did not (P=0.022 and P=0.048, respectively; Figs. 3C and 4C). However, in the luminal and HER2 subtypes, no significant differences were observed in OS or DFS between patients who achieved pCR and those who did not (OS: P=0.24 and P=0.48, respectively; DFS: P=0.56 and P=0.61, respectively; Figs. 3A, 3B, 4A and 4B).

### Prognosis of patients with the HER2 disease subtype who exhibited DFS events

Table II shows four cases of the HER2 subtype with a DFS event. The events in three of the four cases were brain metastasis without any other metastasis. All three of these patients received radiation therapy or surgical resection, and survived without any metastasis other than brain metastasis. The fourth case did not survive due to reasons that were unrelated to the disease.

## Discussion

A higher pCR rate was previously observed when breast cancer patients with ER- and PgR-negative tumors received NAC (11–15). In HER2-positive cases, NAC was more effective than in other cases due to the standard concurrent use of trastuzumab (12–16). In the present study, relatively high pCR rates were achieved among all disease subtypes. The 31% pCR rate observed in the luminal subtype was markedly higher than that in previous studies. Since pretreatment histological grade and Ki-67 index were not assessed in the current study, the luminal A subtype was not accurately distinguished from the luminal B subtype. Therefore, the high pCR rate observed in ER-positive tumors may be explained by the possibility that the majority of the patients in this subgroup exhibited luminal B tumors.

Patients with luminal tumors and HER2-positive tumors exhibited good prognosis in terms of OS. Conversely, the prognosis of patients with TN tumors was significantly poorer than that of patients with other tumor subtypes. Previously, administration of aromatase inhibitors and trastuzumab as standard adjuvant therapy improved the prognosis of patients with luminal and HER2-positive subtypes (17–20). However, the prognosis of patients with TN tumors remains poor as no new therapeutic agents targeting TN tumors are available. Although patients with luminal tumors exhibited a relatively good prognosis in terms of DFS, those with HER2-positive tumors exhibited poor prognosis in comparison. No metastasis, other than brain metastasis, was observed in patients with the HER2 disease subtype. If brain metastasis is ignored, all patients with the HER2 disease subtype exhibited no metastasis and good prognosis.

pCR is generally considered a predictor of OS and DFS. In the present study, pCR following NAC significantly improved OS, but not DFS. The lack of correlation between pCR and DFS may be due to the small size of the study population and since two patients with brain metastasis and one patient who committed suicide were among the patients who achieved pCR. Without the latter three cases, pCR is likely to have been a significant predictor of DFS.

pCR significantly improved OS and DFS only in patients with TN breast cancer. In the luminal subtype, pCR was found to correlate with good prognosis, but the correlation was not statistically significant, possibly due to the small number of events. In the HER2 subtype, NAC was extremely effective, achieving 100% response and 67% pCR rates. In addition, all patients in that subgroup were recurrence-free, with the exception of patients with brain metastasis to whom chemotherapeutic agents and antibodies were not administered. Consequently, HER2-positive patients who received NAC exhibited a good prognosis regardless of whether the patients achieved pCR. In addition, the prognosis of HER2-positive patients who developed brain metastasis may be improved than that of HER2-negative patients (21). Whole brain radiation therapy (WBRT) is a mainstay of treatment for brain metastasis. However, the deterioration of cognitive function due to late radiation toxicity following WBRT is an important issue among long-term survivors (22). If brain metastases are few and small, stereotactic radiotherapy, which is less invasive and more effective than WBRT, may be elected (23). Therefore, further discussion of routine brain screening following surgery, particularly in HER2-positive patients, is required.

Since the design of the current study was retrospective and included a small number of patients, results must be interpreted with caution. Prospective studies incorporating large numbers of patients and longer follow-up periods are required to assess the prognostic value of pCR in patients with initial unfavorable prognoses and to define appropriate therapeutic strategies.

In patients with luminal tumors and HER2-positive tumors, pCR was not useful as a surrogate marker of OS or DFS; other surrogate markers may exist in this subtype since prognosis was affected by hormone and anti-HER2 therapy. In patients with TN tumors, the degree of pathological response following NAC may highlight important prognostic information, as chemotherapy was the only effective therapy for that disease subtype.

## Figures and Tables

**Table I:** Univariate analysis of factors predicting pCR in breast cancer following NAC.

	Patients achieving		
Factors	Patients, n (%)	pCR, n (%)	P-value
Age, years			
≤49	37 (41.1)	16 (43)	0.87
≥50	53 (58.9)	22 (42)	
Stage			
IIA	23 (25.6)	9 (39)	0.50
IIB	40 (44.4)	15 (38)	
III	27 (30.0)	14 (52)	
T factor			
T1	12 (13.3)	9 (75)	0.013
T2–3	78 (86.7)	29 (37)	
N factor			
N0	16 (17.8)	4 (25)	0.058
N1–2	74 (82.2)	34 (52)	
ER status			
Positive	43 (47.8)	13 (30)	0.028
Negative	47 (52.2)	25 (53)	
PgR status			
Positive	38 (42.2)	11 (29)	0.029
Negative	52 (57.7)	27 (52)	
HER2 status			
Positive	21 (23.3)	12 (57)	0.13
Negative	69 (76.7)	26 (38)	
Subtype			
Luminal	48 (53.3)	15 (31)	0.049
TN	27 (30.0)	13 (48)	
HER2	15 (16.7)	10 (67)	
Total	90	38 (42)	

pCR, pathological complete response; NAC, neoadjuvant chemotherapy; ER, estrogen receptor; PgR, progesterone receptor; HER2, human epidermal growth factor receptor 2; TN, triple-negative.

**Table II:** HER2 subtype and DFS events in four cases.

Case	Age, years	T	N	Stage	Subtype	Outcome	Event	DFS, days	OS, days	Survival
1	53	2	1	2B	HER2	PR	Brain metastasis	540	1,621	Survived
2	41	1	2	3A	HER2	CR	Brain metastasis	205	1,299	Survived
3	50	2	2	3A	HER2	CR	Suicide	227	227	Succumbed
4	71	2	1	2B	HER2	CR	Brain metastasis	502	1,009	Survived

HER2, human epidermal growth factor receptor 2; DFS, disease-free survival; T, T factor; N, N factor; OS, overall survival; CR, complete response; PR, partial response.

## References

[1] Wolmark N, Wang J, Mamonas E, Fisher B (2001). Preoperative chemotherapy in patients with operative breast cancer: nine-year results from National Surgical Adjuvant Breast and Bowel Project B-18. J Natl Cancer Inst Monogr.

[2] Bear HD, Anderson S, Brown A (2003). The effect on tumor response of adding sequential preoperative docetaxel to preoperative doxorubicin and cyclophosphamide: preliminary results from National Surgical Adjuvant Breast and Bowel Project B-27. J Crin Oncol.

[3] Smith IC, Heys SD, Hutcheon AW (2002). Neoadjuvant chemotherapy in breast cancer: significantly enhanced response with docetaxel. J Clin Oncol.

[4] Ring AE, Smith IE, Ashley S, Fulford LG, Lakhani SR (2004). Oestrogen receptor status, pathological complete response and prognosis in patients receiving neoadjuvant chemotherapy for early breast cancer. Br J Cancer.

[5] Fasching PA, Heusinger K, Haeberle L (2011). Ki67, chemotherapy response, and prognosis in breast cancer patients receiving neoadjuvant treatment. BMC Cancer.

[6] von Minckwitz G, Untch M, Blohmer JU (2012). Definition and impact of pathologic complete response on prognosis after neoadjuvant chemotherapy in various intrinsic breast cancer subtypes. J Clin Oncol.

[7] Kawajiri H, Takashima T, Onoda N (2012). Efficacy and feasibility of neoadjuvant chemotherapy with FEC 100 followed by weekly paclitaxel for operable breast cancer. Oncol Lett.

[8] Buzdar AU, Ibrahim NK, Francis D (2005). Significantly higher pathologic complete remission rate after neoadjuvant therapy with trastuzumab, paclitaxel, and epirubicin chemotherapy: results of a randomized trial in human epidermal growth factor receptor 2-positive operable breast cancer. J Clin Oncol.

[9] The Japanese Breast Cancer Society (2008). General Rules for Clinical and Pathological Recording of Breast Cancer.

[10] Wolff AC, Hammond ME, Schwartz JN (2007). American Society of Clinical Oncology/College of American Pathologists guideline recommendations for human epidermal growth factor receptor 2 testing in breast cancer. J Clin Oncol.

[11] Keskin S, Muslumanoglu M, Saip P (2011). Clinical and pathological features of breast cancer associated with the pathological complete response to anthracycline-based neoadjuvant chemotherapy. Oncology.

[12] Tan MC, Al Mushawah F, Gao F (2009). Predictors of complete pathological response after neoadjuvant systemic therapy for breast cancer. Am J Surg.

[13] Jones RL, Salter J, A’Hern R (2010). Relationship between oestrogen receptor status and proliferation in predicting response and long-term outcome to neoadjuvant chemotherapy for breast cancer. Breast Cancer Res Treat.

[14] Colleoni M, Bagnardi V, Rotmensz N (2009). Increasing steroid hormone receptors expression defines breast cancer subtypes non responsive to preoperative chemotherapy. Breast Cancer Res Treat.

[15] Toi M, Nakamura S, Kuroi K (2008). Phase II study of preoperative sequential FEC and docetaxel predicts of pathological response and disease free survival. Breast Cancer Res Treat.

[16] Buzdar AU, Valero V, Ibrahim NK (2007). Neoadjuvant therapy with paclitaxel followed by 5-fluorouracil, epirubicin, and cyclophosphamide chemotherapy and concurrent trastuzumab in human epidermal growth factor receptor 2-positive operable breast cancer: an update of the initial randomized study population and data of additional patients treated with the same regimen. Clin Cancer Res.

[17] Forbes JF, Cuzick J, Arimidex, Tamoxifen, Alone or in Combination (ATAC) Trialists’ Group (2008). Effect of anastrozole and tamoxifen as adjuvant treatment for early-stage breast cancer: 100-month analysis of the ATAC trial. Lancet Oncol.

[18] Colleoni M, Giobbie-Hurder A, Regan MM (2011). Analyses adjusting for selective crossover show improved overall survival with adjuvant letrozole compared with tamoxifen in the BIG1–98 study. J Clin Oncol.

[19] van de Velde CJ, Rea D, Seynaeve C (2011). Adjuvant tamoxifen and exemestane in early breast cancer (TEAM): a randomised phase 3 trial. Lancet.

[20] Gianni L, Dafni U, Gelber RD (2011). Treatment with trastuzumab for 1 year after adjuvant chemotherapy in patients with HER2-positive early breast cancer: a 4-year follow-up of a randomised controlled trial. Lancet Oncol.

[21] Wolstenholme V, Hawkins M, Ashley S, Tait D, Ross G (2008). HER2 significance and treatment outcomes after radiotherapy for brain metastases in breast cancer patients. Breast.

[22] Aoyama H (2011). Radiation therapy for brain metastases in breast cancer patients. Breast Cancer.

[23] Matsumoto K, Ando M, Yamauchi C (2009). Questionnaire survey of treatment choice for breast cancer patients with brain metastasis in Japan: results of a nationwide survey by the task force of the Japanese Breast Cancer Society. Jpn J Clin Oncol.

